# Successful percutaneous left atrial appendage occlusion for atrial fibrillation in a patient with mirror-image dextrocardia: a case report

**DOI:** 10.1186/s12872-021-02369-9

**Published:** 2022-01-29

**Authors:** Jizhe Xu, Gaxue Jiang, Lu Zhang, Zixian Chen, Hao Wang, Ming Bai, Jie Zeng

**Affiliations:** 1 Heart Center, Gansu Heart Center, Key Laboratory for Cardiovascular Disease of Gansu, The First Hospital of Lanzhou University, Lanzhou, China; 2 Department of Radiology, The First Hospital of Lanzhou University, Lanzhou, China; 3 Department of Anesthesiology, The First Hospital of Lanzhou University, Lanzhou, China; 4 Department of Cardiology, Sichuan Academy of Medical Sciences and Sichuan Provincial People’s Hospital, University of Electronic Science and Technology of China, Chengdu, China

**Keywords:** Mirror-image dextrocardia, Atrial fibrillation, Percutaneous left atrial appendage occlusion

## Abstract

**Background:**

Dextrocardia is a rare congenital condition (1/10,000–12,000) and AF is uncommon (1–2%). Therefore, the occurrence of the two conditions is rare. Percutaneous left atrial appendage occlusion (LAAO) is a treatment to prevent atrial fibrillation (AF)-associated thromboembolic events.

**Case presentation:**

An 85-year-old female with known situs inversus totalis, persistent AF, and stroke was treated with oral anticoagulation, but she was suffering from constant gingival bleeding. Her CHA2DS2VASc score was 6 points (abnormal, ≥ 2), and her HAS-BLED score was 4 points (abnormal, ≥ 3). The transthoracic echocardiography (TTE) demonstrated left atrial (LA) enlargement (46 mm) and 50% of ejection fraction. She underwent percutaneous LAAO for stroke recurrence prevention using a Watchman occluder. The operation was successful but with technical differences compared with a standard case because of the dextrocardia.

**Conclusion:**

This is the first reported case of a percutaneous LAAO in situs inversus dextrocardia. This case indicates the feasibility of LAAO in congenital cardiac malposition combined with AF.

## Background

Mirror-image dextrocardia is a congenital malposition that occurs in only 1 in 10,000–12,000 humans [1]. In mirror-image dextrocardia, the heart is a mirror image of a normal heart but pointing to the right side [2, 3]. Technically, people with dextrocardia do not have special health disorders and usually have no symptoms, but they can be prone to some disorders of the bowels, esophagus, bronchi, and blood vessels [2, 3].

Atrial fibrillation (AF) is a common supraventricular tachyarrhythmia caused by uncoordinated atrial activation and associated with an irregularly irregular ventricular response [4]. The causes of AF include underlying structural heart disease, metabolic disorders, endocrine diseases, and certain medications [5]. The prevalence of AF is approximately 1–2% in the general population of developed countries [5]. Patients with AF are often at a significantly increased risk of thromboembolism and, in particular, stroke [4, 6].

AF, in general, is thought to occur as often in patients with dextrocardia as in the general population. So, given the rarity of the malposition and the low frequency of AF, AF seen in this special population is extremely rare. This paper reports the case of a patient with dextrocardia and AF who underwent percutaneous left atrial appendage (LAA) occlusion (LAAO) successfully.

## Case presentation

A Han 85-year-old female with known mirror-image dextrocardia presented in January 2018 to the Emergency Room of the Second Hospital of Lanzhou University due to dizziness and aphasia. The electrocardiogram (ECG) showed a definitive diagnosis of AF, and she was diagnosed with stroke based on clinical symptoms, neurological physical examination, and brain magnetic resonance imaging (MRI). The neurologist gave her optimal medical therapy (anticoagulation, antioxidants, plaque stabilization, and rehabilitation therapy), and she recovered well. The neurologist considered that the stroke was due to AF thrombosis, and the patient accepted warfarin 3 mg/day for oral anticoagulation (OAC). She underwent international normalized ratio (INR) monitoring after discharge.

Subsequently, the patient developed persistent gum bleeding on warfarin OAC for 6 months. She had a 10-year history of hypertension but with good control using oral valsartan 80 mg/day and amlodipine 5 mg/day. In the physical examination, she showed normal vital signs and a body mass index (BMI) of 15.6 kg/m^2^. The heart border could be seen on the right side of her chest. Heart sounds could be auscultated, but she displayed weak S_1_ and irregular beats without any murmurs or peripheral edema.

**Fig. 1:**
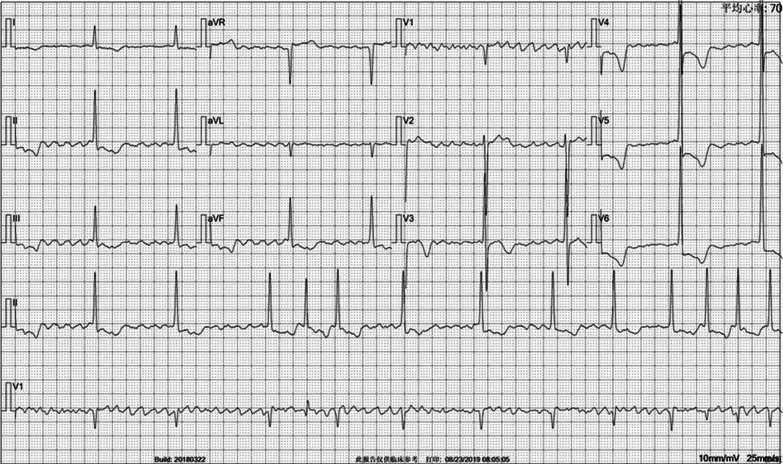
ECG showing atrial fibrillation rhythm. The average heart rate was 70 bpm. The ST segment was depressed. T wave inversion was observed on the II, III, aVF, and V3-V6 leads by the corrected examination

**Fig. 2:**
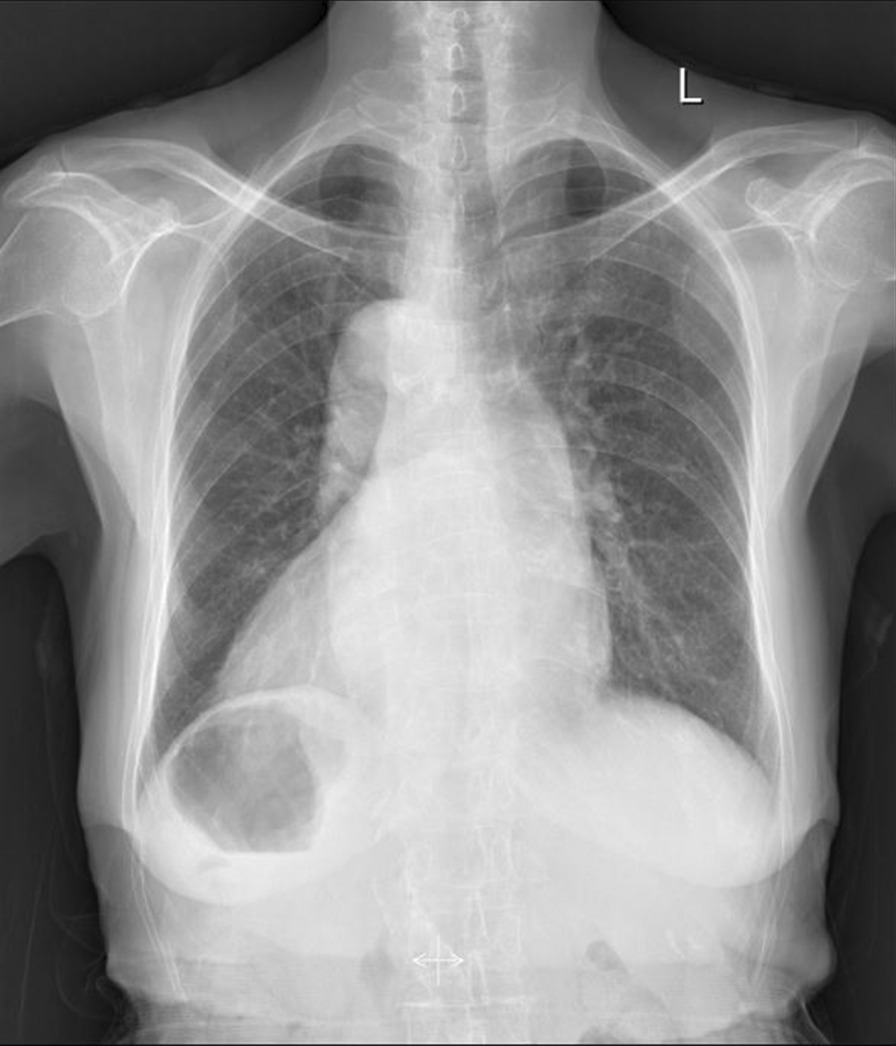
Chest X-ray showed mirror-image dextrocardia, with the heart’s silhouette on the right side of her chest

Her CHA2DS2VASc score was 6 points (abnormal, ≥ 2), and her HAS-BLED score was 4 points (abnormal, ≥ 3). The laboratory assay INR was 3.6 (normal range 0.86–1.13). ECG showed AF (Fig. 1). Chest X-ray (CXR) showed the heart on the right side of her chest (Fig. 2). The transthoracic echocardiography (TTE) demonstrated left atrial (LA) enlargement (46 mm) and 50% of ejection fraction.

**Fig. 3:**
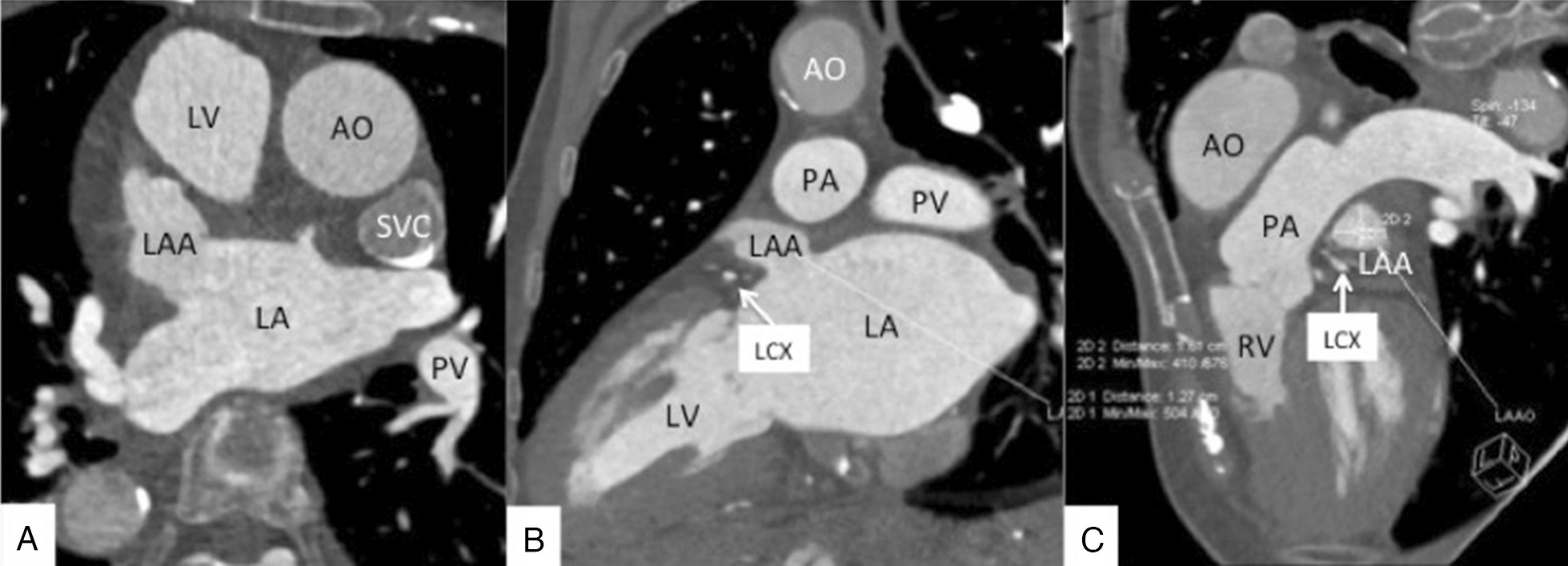
Computed tomographic angiography of the LA and LAA showing mirror dextrocardia. The LA and LAA had no filling defect in early- and late-phase images (**A**, **B**). Reconstruction images showing that the LAA ostial was 16 mm; the depth of the LAA was 19 mm (**C**). *AO* aorta, *LA* left atrium, *LAA* left atrial appendage, *LV* left ventricular, *PA* pulmonary artery, *PV* pulmonary vein, *LCX* left circumflex artery, *SVC* superior vena cava

Two-phase cardiac computed tomographic (CT) angiography of the LA and LAA showed mirror dextrocardia without filling defects in LA and LAA on both the early- and late-phase images (Fig. 3A, B). Reconstruction images showed the ostial and depth of the LAA (Fig. 3C).

Because of the limitations of OAC, and in particular bleeding events occurring after standard anticoagulant therapy, she was deemed with contraindication to OAC. LAAO was performed under general anesthesia successfully.

**Fig. 4:**
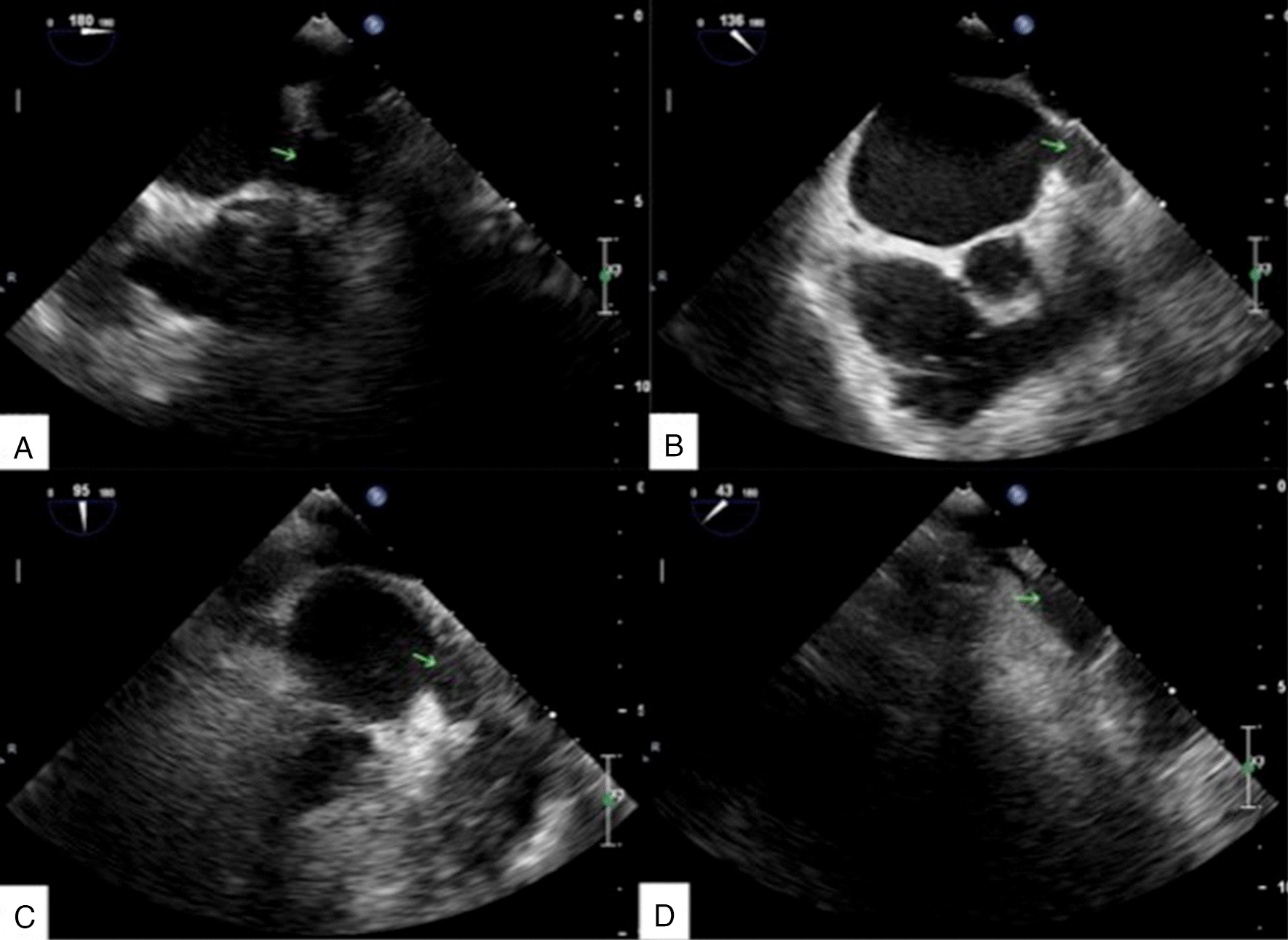
Transesophageal echography (TEE) images in the corrected angles of 0° (**A**), 45° (**B**), 90° (**C**), and 135° (**D**). The arrows show the left atrial appendage, clean without thrombosis

After anesthesia, the echocardiography specialist inserted the transesophageal echography (TEE) probe to check for thrombosis and LAA evaluation. For the examination, the TEE probe was rotated slightly to the right side of the patient. The heart images were completely mirrored and symmetrical. When observing the LAA at different angles, it was also axisymmetric with the common angle. Therefore, to display the internal structure of the LAA in this case, the angle pointer of the probe chip was turned clockwise from 180° to 45°, and the internal part of the LAA was scanned continuously. The usual images at 0°, 45°, 90°, and 135° were displayed at 180°, 135°, 90°, and 45° in this case (Fig. 4A–D).

**Fig. 5:**
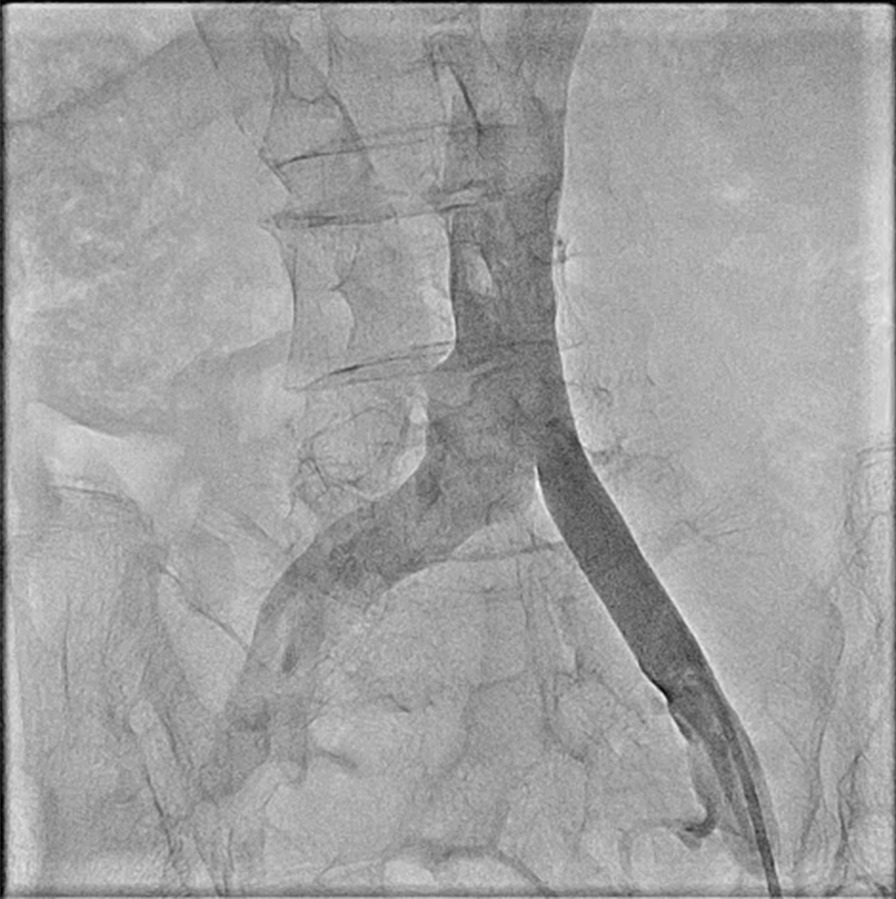
Pigtail catheter verification of the iliac vein by angiography

The vascular access was established by the left femoral vein, and an 8F sheath was inserted. Due to the dextrocardia, the iliac vein was checked by angiography using a pigtail catheter. The angiography showed that the left iliac vein was straighter than the right (Fig. 5).

An 8F Swartz sheath was exchanged via its wire to the superior vena cava (SVC). A transseptal needle was inserted into the Swartz sheath. TEE guidance was used for the paracentesis of the atrial septum. The TEE angle of the long axis of the atrial septum was about 60°, and it was axisymmetric with that of common cases. Furthermore, the image of the short axis of the atrial septum was about 115° by X plane function.

**Fig. 6:**
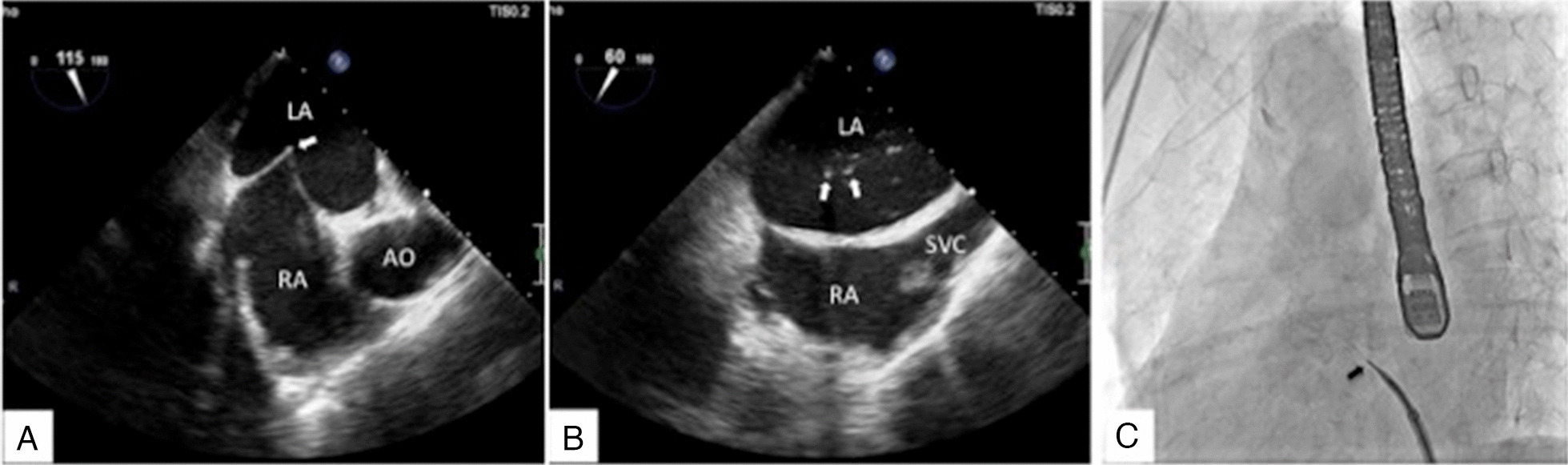
A Swartz sheath was pushed to the atrial septum (the arrow in **A**). Injection of saline showed the bubble sign after puncturing the atrial septum (the arrows in **B**). The contrast agent is linear after successful septum puncture by fluoroscopy (the arrow in **C**). *AO* aorta, *LA* left atrium, *RA* right atrial, *SVC* superior vena cava

Using TEE and fluoroscopy, the Swartz sheath was withdrawn and glided to the low order of the atrial septum, turned anticlockwise, checked by TEE, and the temping sign was observed (Fig. 6A). The point of puncture was low order and retroposition of the septum. The septum was smoothly punctured, checked by TEE and fluoroscopy (Fig. 6B, C). Unfractionated heparin (UFH) (4000 U) was injected immediately via a peripheral vein and the activated clotting time (ACT) was 302 s 10 min later.

**Fig. 7:**
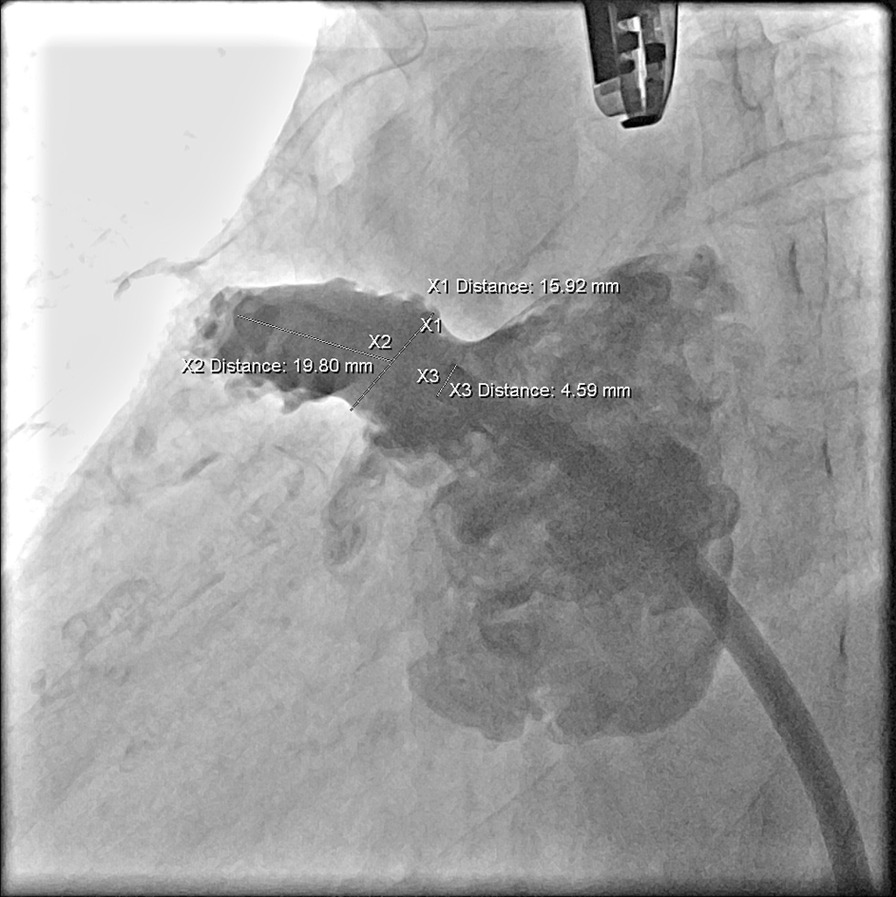
Pigtail catheter for left atrial appendage (LAA) angiography to check the opening and depth of the LAA

The needle was withdrawn, and the wire was exchanged to the top left corner pulmonary vein. An Amplatz super-stiff wire was exchanged to the top left corner pulmonary vein by the Swartz sheath. The Watchman left appendage occluder device transducer system was conveyed into the LA, carefully eliminated the bubbles from the system, and added a pigtail catheter into the transducer. The ring of the pigtail was placed into the LAA for angiography. The wing’s tips pointed right, and the opening of the LAA was 16 mm (Fig. 7).

**Fig. 8:**
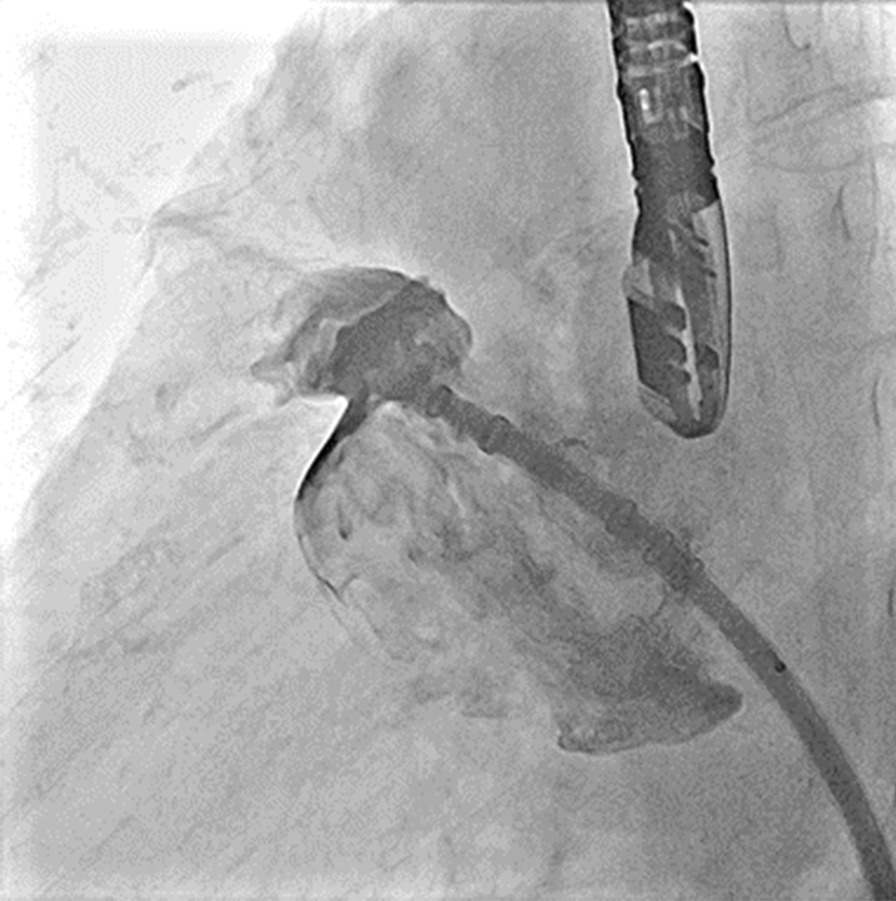
Angiography for left atrial appendage occlusion (LAAO) after device implantation, showing no residual leakage and complete occlusion

**Fig. 9:**
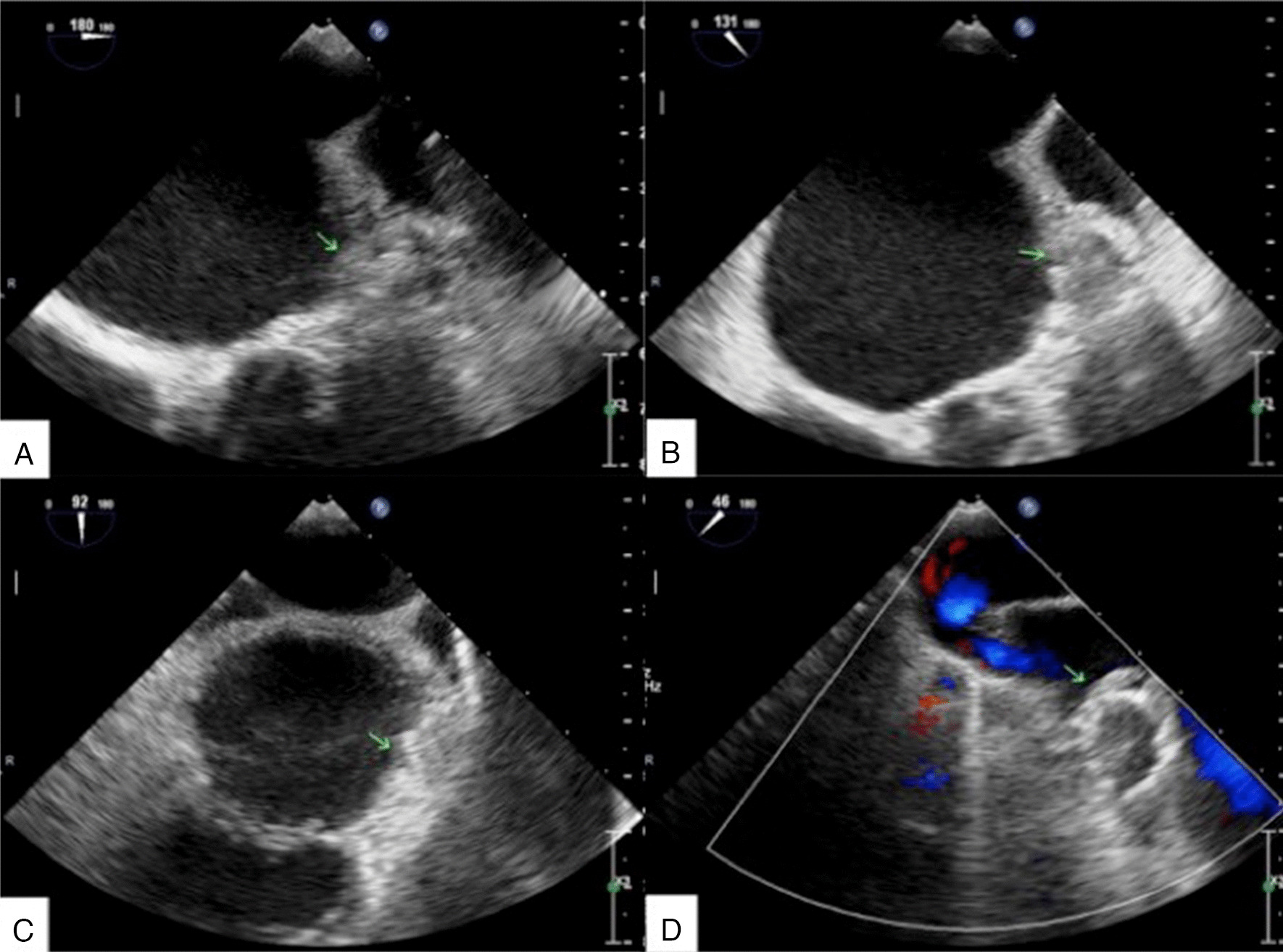
Verification at different angles after Watchman device implantation. Transesophageal echography (TTE) showed that the device was implanted in a favorable position without residual leakage. Following the PASS principle (position, anchor, seal, and size), the stability was tested after the device was implanted. The ratio of compression was 20–23%

The axial direction of the transducer sheath was adjusted, and a 21-mm Watchman device was implanted carefully. The device was checked by TEE and angiography (Figs. 8, 9). The device was liberated, and the transducer was removed. TEE was used to check the geometry of the LAA and any pericardial effusion again. The transducer was withdrawn, and the operation was completed. The total operation time was 139 min, and the total amount of contrast agent (iopromide 370) was 160 ml.

There were no clinical signs of pericardial effusion and no significant elevation of the cardiac biomarkers. The patient reported no symptoms and was discharged on the third day after the procedure with aspirin 100 mg/day and clopidogrel 75 mg/day for dual anti-platelet aggregation therapy for 6 months. The patient’s postoperative course was uneventful.

**Fig. 10:**
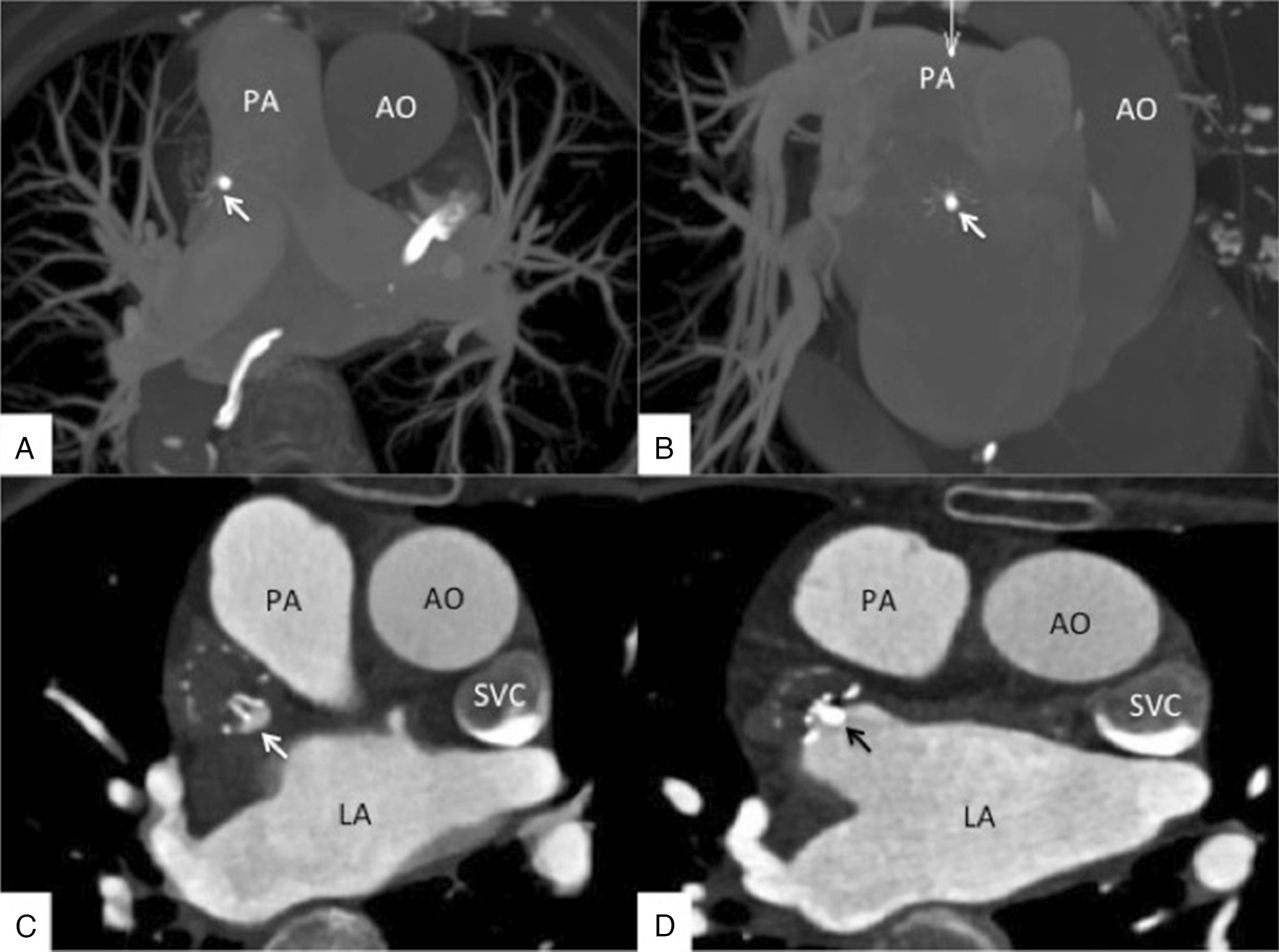
The arrows show that the Watchman device was implanted in a favorable position. No contrast agent was developed at the LAA. No device-related thrombosis and pericardial effusion were observed. *AO* aorta, *LA* left atrium, *LAA* left atrial appendage, *PA* pulmonary artery, *SVC* superior vena cava

**Fig. 11:**
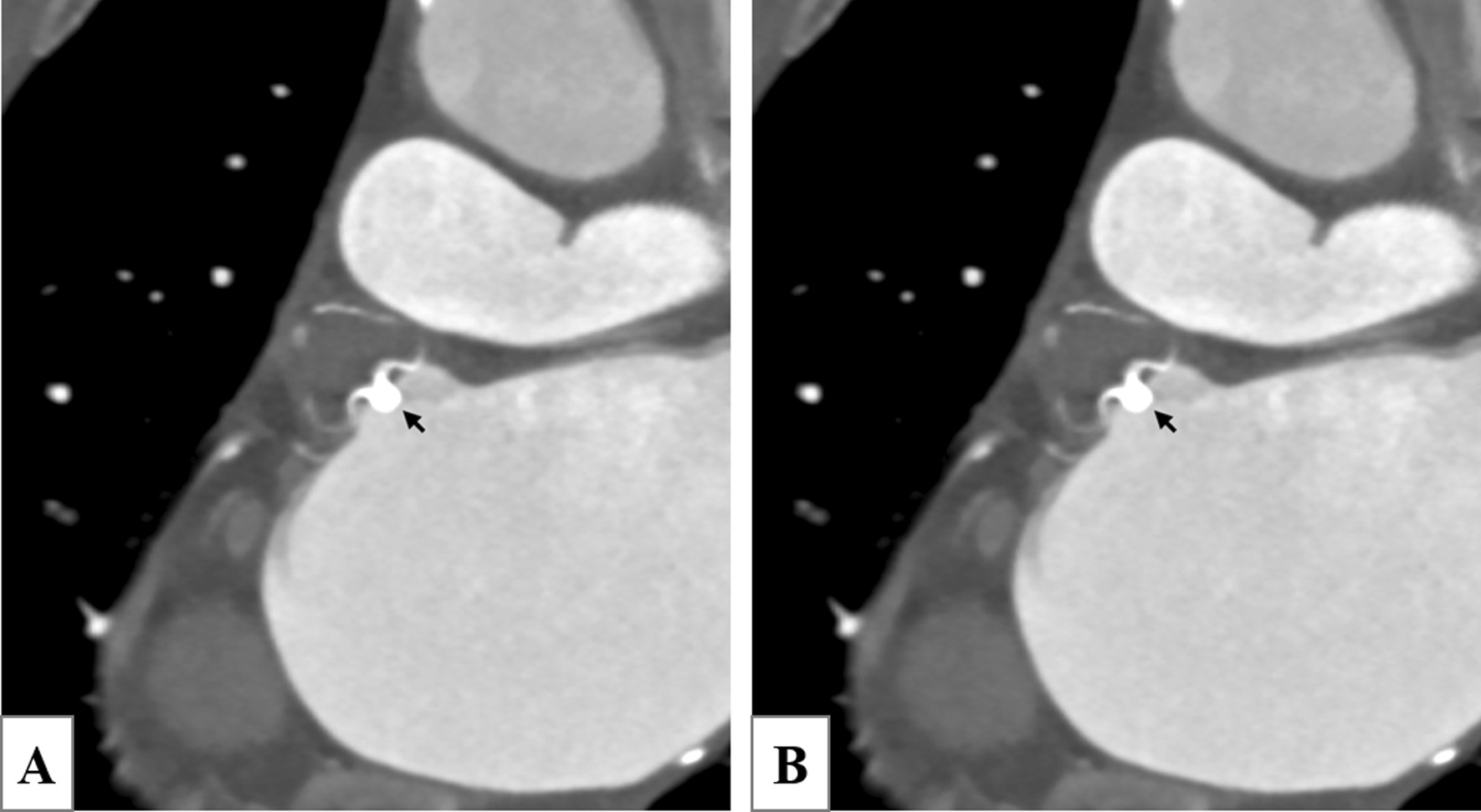
CTA at 6 months shows that the long axis of the sealing device fits well with the long axis of the orifice. There is no contrast agent leakage and no contrast agent filling in the sealing device, indicating that the sealing is successful and the effect is good (**A**). The arrow shows the short axis of the auricular orifice. CTA shows that the long axis of the sealing device fits well with the anchoring area of the auricular orifice. There is no thrombosis on the surface of the device, and the sealing effect is good (**B**)

She completed her 45-days follow-up on October 8, 2019. She reported no symptoms. CT angiography follow-up was performed (Fig. 10). CTA images showed that the patient was successfully blocked, and the effect was good (Fig. 11).

## Discussion

Dextrocardia is a rare congenital condition (1/10,000–12,000) [1] and AF is uncommon (1–2%) [5]. Therefore, the occurrence of the two conditions is rare. This paper presented the case of a woman with both conditions in whom percutaneous LAAO was successfully performed.

AF is associated with stroke, and anticoagulation drugs and LAAO are common treatments to prevent a first stroke or a recurrence [4, 6]. The PROTECT AF and PREVAIL studies provided evidence for the protective effect of LAAO on thromboembolic event prevention [4, 6]. Especially in patients with high CHA2DS2VASc and HAS-BLED scores, the use of anticoagulation is limited, and LAAO is the procedure of choice [4, 6].

Due to growing experience with the percutaneous interventional approach for LAAO, the procedural success rates for LAAO have significantly improved within the last years and are now considered within the 98% range [7, 8]. In experienced centers, it can nowadays routinely be performed.

**Fig. 12:**
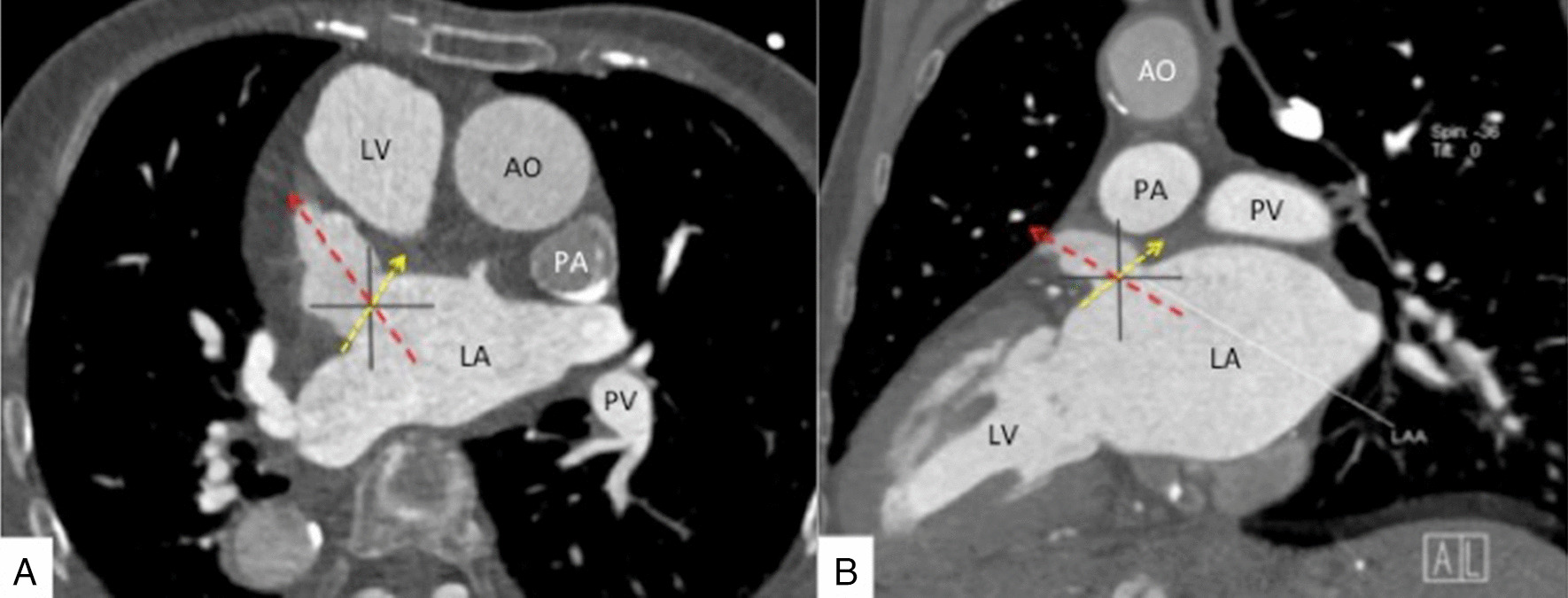
Preoperative computed tomography (CT) guidance. The yellow arrows show the ostium of the left atrial appendage (LAA). The red arrow shows the macro axis of the LAA. The angle of the angiography projection was according to the CT guidance. The best projection angle was LAO 46° + CREA 23°. *AO* aortic, *LA* left atrial, *PA* pulmonary artery, *SVC* superior vena cava

Dextrocardia is rare in the general population [1] and can be associated with significant additional cardiac malformations [2, 3, 9]. AF, in general, occurs as often in patients with dextrocardia as in the general population. Still, the changes in the structure of the heart can be confusing. The key to the success of LAAO in dextrocardia is to analyze the image data of the patients before the operation and make the strategy. CTA could give the optimal angles of projection (Fig. 12A, B). It will be necessary to find access favorable for the operation, adapt to the opposite procedure, and choose the image reversal function of the digital subtraction angiography machine if necessary. These points are also highlighted in various percutaneous heart interventions in dextrocardia, including catheter ablation of paroxysmal focal AF [10–12] and LAAO [13–15].

## Conclusion

Mirror dextrocardia is a rare congenital malformation, and dextrocardia with AF is even rarer. This paper presented one case of mirror dextrocardia who underwent LAAO. Combined with the clinical information provided by CTA, the judgment, surgical approach, preoperative DSA pitch angle, and TEE approach was modified according to the patient’s condition, resulting in successful LAAO. The authors’ presurgical mirror planning was confirmed intraoperatively.

## Data Availability

The datasets used and/or analyzed during the current study are available from the corresponding author on reasonable request.

## Abbreviations

LAAO: Left atrial appendage occlusion
AF: Atrial fibrillation
TTE: The transthoracic echocardiography
LA: Demonstrated left atrial
ECG: The electrocardiogram
MRI: Magnetic resonance imaging
OAC: Oral anticoagulation
INR: International normalized ratio
BMI: Body mass index
CXR: Chest X-ray
CT: Computed tomographic
TEE: The transesophageal echography
SVC: Superior vena cava
UFH: Unfractionated heparin
ACT: Activated clotting time
